# Novel single stage multipath 8 × 8 interconnection network/switching element (SSMIN/E) architecture

**DOI:** 10.1038/s41598-026-48611-2

**Published:** 2026-04-18

**Authors:** Shilpa Gupta, Madhu Bala, Satish R. Jondhale, Amruta S. Jondhale, Geetanjali Sharma

**Affiliations:** 1 Department of Electronics and Communication Engineering, Chandigarh University, Mohali, Punjab, India; 2 Department of Computer Science and Engineering, Lovely Professional University, Phagwara, Punjab India; 3 Amrutvahini COE, Sangamner, Maharashtra India; 4 Pravara Rural Education Society, Loni, Maharashtra India; 5 Symbiosis Institute of Technology, Pune Campus, Symbiosis International (Deemed University), Pune, India

**Keywords:** Switching Elements (SEs), Single Stage Multipath Interconnection Network/Element (SSMIN/E), Gamma interconnection network (GIN), Shuffle Exchange Networks (SEN), Reliability, Fault Tolerance, Engineering, Mathematics and computing

## Abstract

Efficient and reliable interconnection networks are fundamental for maintaining performance in contemporary high-speed parallel computing systems. Traditional 8 × 8 switching elements (SEs) commonly employed in such networks provide only one fixed path between each input-output pair. This absence of redundancy results in single points of failure, making the whole system less reliable. To address the limitation discussed, this manuscript proposes a novel 8 × 8 full crossbar SE with intrinsic path redundancy. The structure employs four 3 × 3 SEs, implemented with eight 2 × 1 multiplexers and 1 × 2 demultiplexers each, to create a Single Stage Multipath Interconnection Network/Element (SSMIN/E). The proposed structure provides two parallel paths between each source-destination pair which improves fault tolerance without increasing routing complexity. The structure is motivated by Gamma interconnect network (GIN), which are appreciated for their scalable and regular structures but are limited by the single-path constraint under the SEs they utilize. The developed SSMIN/E overcomes this limitation by incorporating redundancy at the structural level. For analysis and validation, the reliability of the proposed 8 × 8 SSMIN/E architecture is compared to traditional shuffle exchange networks (SEN) and gamma networks with their newer architectural extensions. The results indicate that the proposed SSMIN/E architecture provides improved reliability and reduced path length, and it has the potential to be an effective solution for fault-tolerant interconnects in high-performance computing applications.

## Introduction

The quick pace of advancement in communication technologies, especially Broadband Integrated Services Digital Networks (B-ISDN), has profoundly impacted the architecture and functionality of contemporary packet-switched networks^1–3^. These trends have fuelled the need for high-speed, integrated communication infrastructure in support of parallel and distributed computing systems. In these systems, several processors work in conjunction with distributed memory modules to efficiently and simultaneously resolve complicated tasks^4,5^. Underlying this computational model are Multistage Interconnection Networks (MINs), which are a structured network that enables effective data transfer between memory modules and processors. In contrast to fully connected crossbars, which provide direct point-to-point communication at high hardware cost, MINs are a cost-effective and scalable means. A typical MIN consists of several stages, with each stage containing SEs that direct data packets according to destination information provided within itself. These SEs interpret routing tags and route packets along suitable outputs, maximizing traffic flow within the network^6^.

Because of their parallel and deterministic routing, MINs surpass bus-based systems in bandwidth utilization and latency, making them ubiquitous for use in supercomputing, grid computing, cloud data centres, and high-end signal processing applications^7–10^. For these applications, many regular compositions of MINs have emerged as a strong candidate for constructing higher order SEs (8 × 8). Gamma networks have gained prominence because of their systematic and fault-tolerant design^11–13^. The Gamma network is a member of the PM2^I^ (plus-minus 2i) family of interconnection networks with a regular, very redundant structure that is particularly suitable for broadband packet switching and high-performance computing applications^14,15,17–20^. The connection pattern between consecutive stages is based on Plus Minus 2^i^ (PM2^i^) pattern, where ‘i’ is the number of stages ranging from ‘0’ to ‘n’. N×N size of PM2^i^ network has ‘N’ number of input and output terminals with ‘n + 1’ number of stages, where ‘n’ = log_2_N. Each stage consists of ‘N’ SEs of configuration 3 × 3 at intermediate stage whereas 1 × 3 and 3 × 1 SEs at input and output stage respectively. Each SE has three input/output stages, where the output terminals of SE at stage ‘i’ are connected to three SEs at stage’ ‘j’ with the connection pattern of ‘(j-2^i^) mod N’, ‘j’ and ‘(j + 2^i^) mod N’. As every stage in a Gamma network consists of 3 × 3 Switching Elements (SEs), and packet routing is based on a tag value: Tag Value ‘T’= [(D-S) mod N].

This value steers the packet to the upper (T = − 1), middle (T = 0), or lower (T = + 1) output port, producing a deterministic, non-blocking route choice^19–21^. Although the Gamma network has numerous benefits—such as fault tolerance, rule-based routing, and parallelism support—it also has challenges. In particular, cases in which all tag values are zero (i.e., source and destination are the same) the network collapses due to a unique-path format with fewer redundancies. In addition, Gamma networks employ larger SEs of size 3 × 3 for each stage, demand N SEs per stage and *N* + 1 stages per network configuration, tremendously escalating hardware complexity for an N×N network. Another class of network which has been extensively studied is Shuffle-Exchange Networks (SENs) with low 2 × 2 SEs accomplish routing with less hardware in the form of only log₂N stages and N/2 SEs per stage^22–25^. Though it also possesses unique path problem between every source-destination pair. Several advancements in SEN topology have been proposed in literature to solve this problem. To overcome the trade-offs among fault tolerance, path length, and hardware efficiency, researchers have proposed variants of SEN such as the SEN-Minus, Pars Network, Extra Group Network (EGN), and Improved EGN (IEGN), each offering one or more improvements^26–29^. Although such efforts are made, it is still an open problem to find a network topology that has low latency, low hardware overhead, and high fault resilience at the same time.

This leads us to propose a novel interconnection architecture: the SSMIN/E. The increasing demands of modern interconnect systems require compact topologies that combine minimum path length, hardware efficiency, reliability, and fault tolerance. In recent literature there are several enhancements to MINs which have been introduced by the researchers to address these needs. For example, a three-layer SiN on optical switch of Si 8 × 8 has been introduced in which the author integrates thermo-optic and electro-optic actuators for high-speed performance^30^ whereas, a compact 8 × 8 electro-optic switch matrices with silicon PIN waveguides has also been introduced recently^31^. The BNRO network which employs reversed outputs to improve routing scalability in NoC-based systems is also an example where large switching elements are required^32^. Furthermore, a replicated multistage MIN design focused on improving quality of service (QoS) in parallel computing by balancing redundancy, resource utilization, and reliability under fault conditions specifically focusses on the design of 8 × 8 SE^33^. The major contributions of this research are as follows:

1. We have proposed novel SSMIN/E architecture which is specifically tailored around an 8 × 8 configuration and introduces a single-stage topology that requires only four 3 × 3 SEs, eight 2 × 1 MUXs, and eight 1 × 2 DEMUXs, offers two disjoint parallel paths for each source–destination pair, and improves fault tolerance with minimal path length and hardware overhead,
2. The 8 × 8 network size is a significant benchmark in current hardware designs and is the fundamental switching granularity in several real-world systems such as Latest optical switching technologies, Replicated MINs optimized for QoS, BNRO architectures for NoC systems, Intel’s 80-tile NoC, IBM’s Blue Gene/L interconnect, and Calient’s photonic switches^30–36^. By employing this size in the proposed SSMIN/E architecture, we not only conform to standard benchmarks but also make it pertinent for actual deployment environments in supercomputers, routers, and broadband.
3. The proposed SSMIN/E architecture illustrates dual-path routing per source-destination pair with increased reliability, minimized path length, and decreased number of components. It offers comparative reliability and QoS analysis with Gamma, SEN, BNRO, and replicated MIN architectures, as well as covers scalability extensions to higher orders (16 × 16 and higher), illustrating how modular replication maintains routing integrity. We have verified performance of the proposed SSMIN/E architecture through analytical modelling and structural comparisons, illustrating its benefits for HPC, NoC, and broadband networks.

The remainder of this paper is outlined as: the Sect.  2 discusses the related research works in the literature, whereas the Sect.  3 describes the proposed SSMIN/E architecture in detail. The Sect.  4 brief the experimental results, followed by conclusion and future scope in the Sect.  5.

## Related work

Gamma network^19^ characterizes a significant development in MINs, contributing redundancy and fault tolerance in the network through multiple paths between all source destination node pairs. Since its inception in 1984, the Gamma network has been a major area of research aimed at improving its reliability and performance for high-speed computing systems^26–29^. It belongs to PM2^i^ class (also known as Data Manipulator networks), is a class of regular multistage networks. In these networks number of input and output ports as well as the number of SEs per stage are equal. Each stage in these networks is characterized by some predefined rules, offering a structured approach to interconnected PEs to the corresponding memory unit to facilitate reliable and efficient communication^37–40^. The configuration of SEs in gamma network consists of 3 × 3 SEs at intermediate stages, while 1 × 3 and 3 × 1 SE configuration is employed at input and output stages, respectively. In gamma network the number of paths between each S-D node pairs are ensured by the tag value ‘T’, which can be calculated using the formula ‘[(D-S) mod N]’, where ‘D’ stands for the destination address, ‘S’ stands for the source address, and ‘N’ is the size of network or number of inputs/outputs of the network. Although gamma network offers innate redundancy and fault tolerance capabilities, but still, there are some challenges^41–43^, particularly for tag value ‘T = 0’, in case of identical source destination address which result in a unique path behaviour, limiting fault tolerance and reliability. This is true in case of shuffle exchange network as well. As this network is also unique path MIN. To address this challenge, researchers have proposed many improved topologies with enhanced features^44^. These enhancements mainly focused on altering connection patterns, introducing extra stages, and using bigger sized SE configurations.

The literature classifies reliability enhancements for Gamma Interconnection Networks (GIN) and Shuffle Exchange Networks (SEN) into several distinct topological and routing categories^45,46^. The first research efforts established network redundancy improvements through two methods which included building additional network stages for EGIN and extra-stage SEN and creating multiple interconnection pathways between source and destination points^16,22^. Other structural approaches involve providing extra links via higher-sized switching elements (SEs)^47–50^ or increasing the number of input/output ports to ensure that critical applications maintain data flow through inherent partial redundancy^]^. The latest research focuses on enhancing both SEs and total stage performance. The implementation of disjoint SE configurations through 3-DGIN and 4-DGIN systems has achieved better fault protection results^21^. The SEN-Minus and Gamma-Minus architectures demonstrate a significant trend, which reduces total stage counts through the implementation of multiplexers (MUXs) and demultiplexers (DEMUXs)^9,11^. The methods FTGM and RGN give priority to MUX/DEMUX components because they achieve higher system reliability through their basic switching components, which have lower failure rates compared to conventional SEs^20,55^. Our proposed SSMIN/E builds on this evolution by integrating 3 × 3 SE subunits to achieve intrinsic redundancy without the overhead of additional stages.

Although these many improvements have been suggested in existing literature but still there is a need to improve the gamma and shuffle exchange network for increasing demands of high-speed computing. Some areas for optimization are as follows:

- Path length optimization is an important method to improve the performance and efficiency. A thorough exploitation of existing network topologies are required to minimize path length while maintaining fault tolerance capability and scalability, thereby reducing latency and improving network throughput. Though as already discussed few topologies have been proposed in past which suggest lowering number of stages to improve path length^44^. But these topologies only reduce one stage and hence, there is a need to exploit the same method to reduce more than one stage to reduce pathlength comprehensively.
- As far as path length is concerned, in MINs, path length usually corresponds to the number of SEs that a data packet has to travel from source to destination node^12,14^. This value directly affects latency, queuing of data, and complexity of routes. Significantly, Only the SEs are taken into account for path length calculation.
- MUX/DEMUX elements only perform signal selection and routing and do not introduce processing delay. In traditional MIN topologies like gamma, there are several SE stages through which the packet has to travel, usually between.
- log_2_N stages, to arrive at the destination^15^. Even for optimized MINs such as SEN or EGN, the minimum path length is typically log_2_N-1. With increasing network size, number of intermediate stages also increases, and thus the path size increases proportionately. On the other hand, the proposed SSMIN/E architecture is structured with a flat, one-stage SE structure, where every source-destination (S–D) pair has two disjoint direct paths through only a single 3 × 3 SE. The network uses MUXs and DEMUXs at the input/output ports for selecting and routing, but these are not counted in the path length since they do not buffer or process packets, hence, do not introduce routing delays simply guide the packet to/from the appropriate SE.
- SE minimization techniques can also be further exploited to lower down the network topologies hardware complexity thereby improving the scalability^29^. A focussed effort can be attempted to develop a topology design strategy that minimizes the number of SEs at each stage while maintaining performance and fault tolerance requirements.

Thus, in sight of the above-mentioned research limitations, there is a long-felt need of industry to address these deficiencies and shortfalls. By addressing these shortfalls and continuing to improve existing topology design, routing algorithms, and fault tolerance mechanisms, the capabilities of gamma networks can further be enhanced and pave the way for their widespread use in real time communication systems.

## Proposed single stage multipath interconnection network (SSMIN/E) architecture

**Fig. 1:**
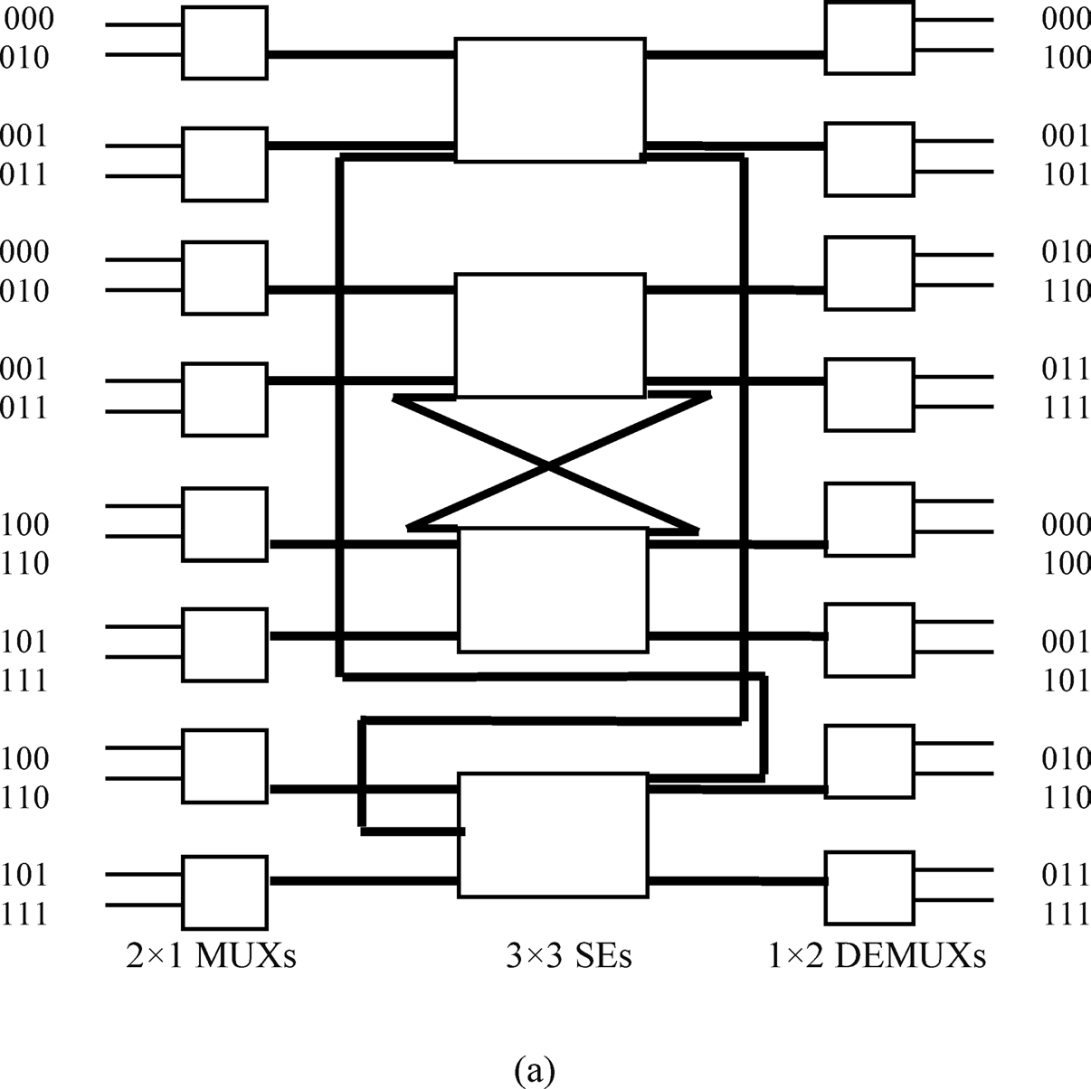

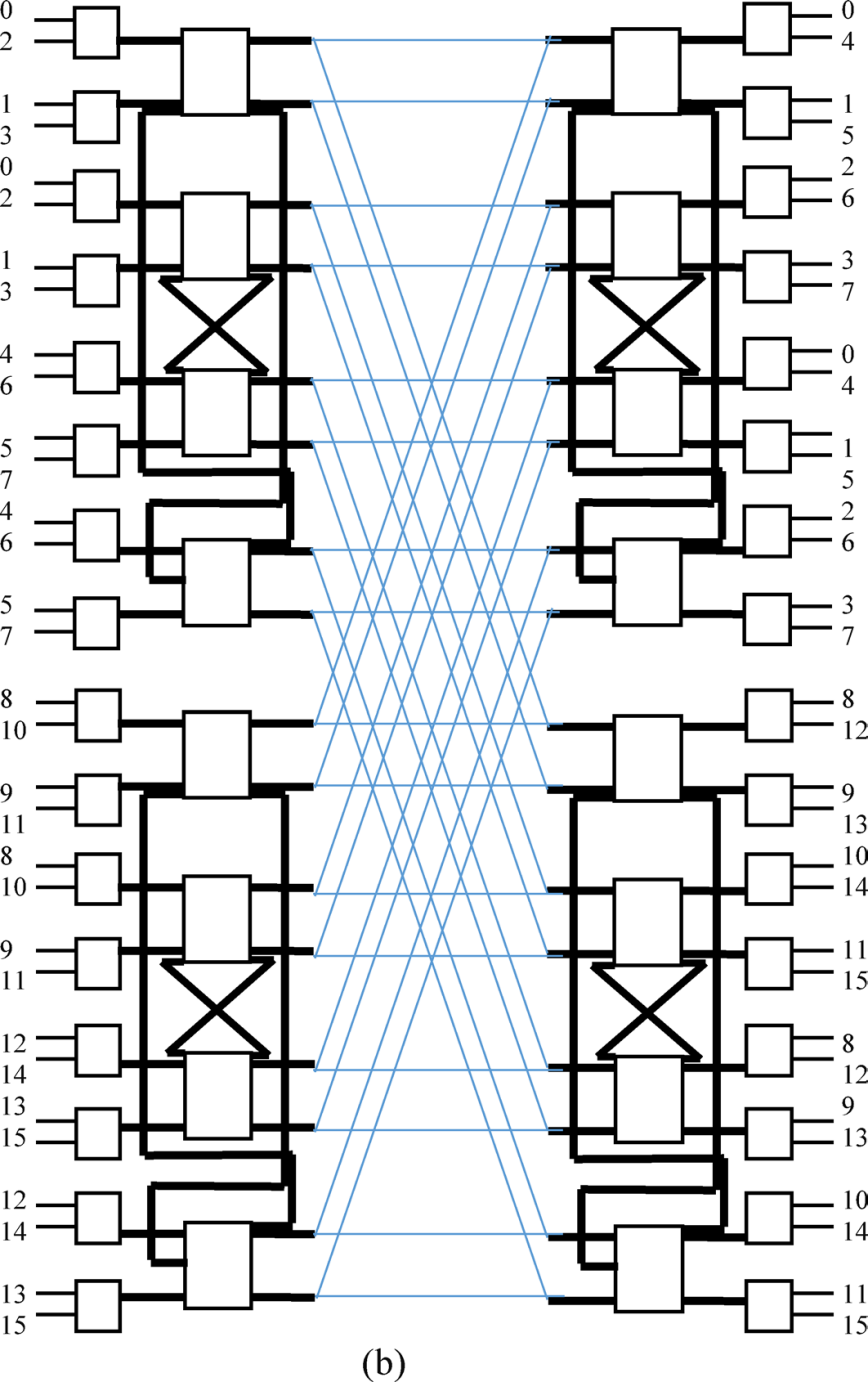
Architecture of the proposed SSMIN/E for (**a**) 8 × 8 and (**b**) 16 × 16 network sizes.

The proposed SSMIN/E architecture is designed for efficient data transmission rates and scalability for complex computing environments. The proposed architecture of size 8 × 8 is composed of ‘4’ 3 × 3 fully cross-bar SEs. These SEs are integrated with 8 MUXs and 8 DEMUXs at input and output terminals respectively, which play critical roles in managing data flow from input to output port and creating parallel terminal paths. The network architecture of proposed SSMIN/E architecture is shown in Fig. 1. In SSMIN/E each 3 × 3 SE consists of 3 input terminals and 3 output terminals. These SEs are capable of routing data dynamically from random input port to any other random output port. Strategically eight MUXs and eight DEMUXs are connected to these fully crossbar SEs. The configuration of these MUXs and DEMUXs is 2 × 1 and 1 × 2 respectively. The MUXs are connected to the input ends of the SEs and DEMUXs are connected to the output ends of the SEs. As suggested in the literature 2 × 1 MUX comprises of 2 input lines and one output line, allowing it to choose between these 2 inputs and put forward the selected input to the output line. The connection pattern between these MUXs and DEMUXs with fully cross-bar SEs and connection of SEs among themselves through chaining enables the formation of multiple parallel paths within the network from each S-D node pair. These multiple paths ensure efficient data flow from source to destination without bottlenecks even in the presence of faults. One of the most important performance indicators of this design architecture is its scalability. This 8 × 8 SSMIN/E can be used as fully cross-bar 8 × 8 SE and can be scaled up to any number of input and output for higher sized network. This scaling capability of proposed SSMIN/E architecture is critical for real time networks that require to handle huge amounts of data.

**Fig. 2:**
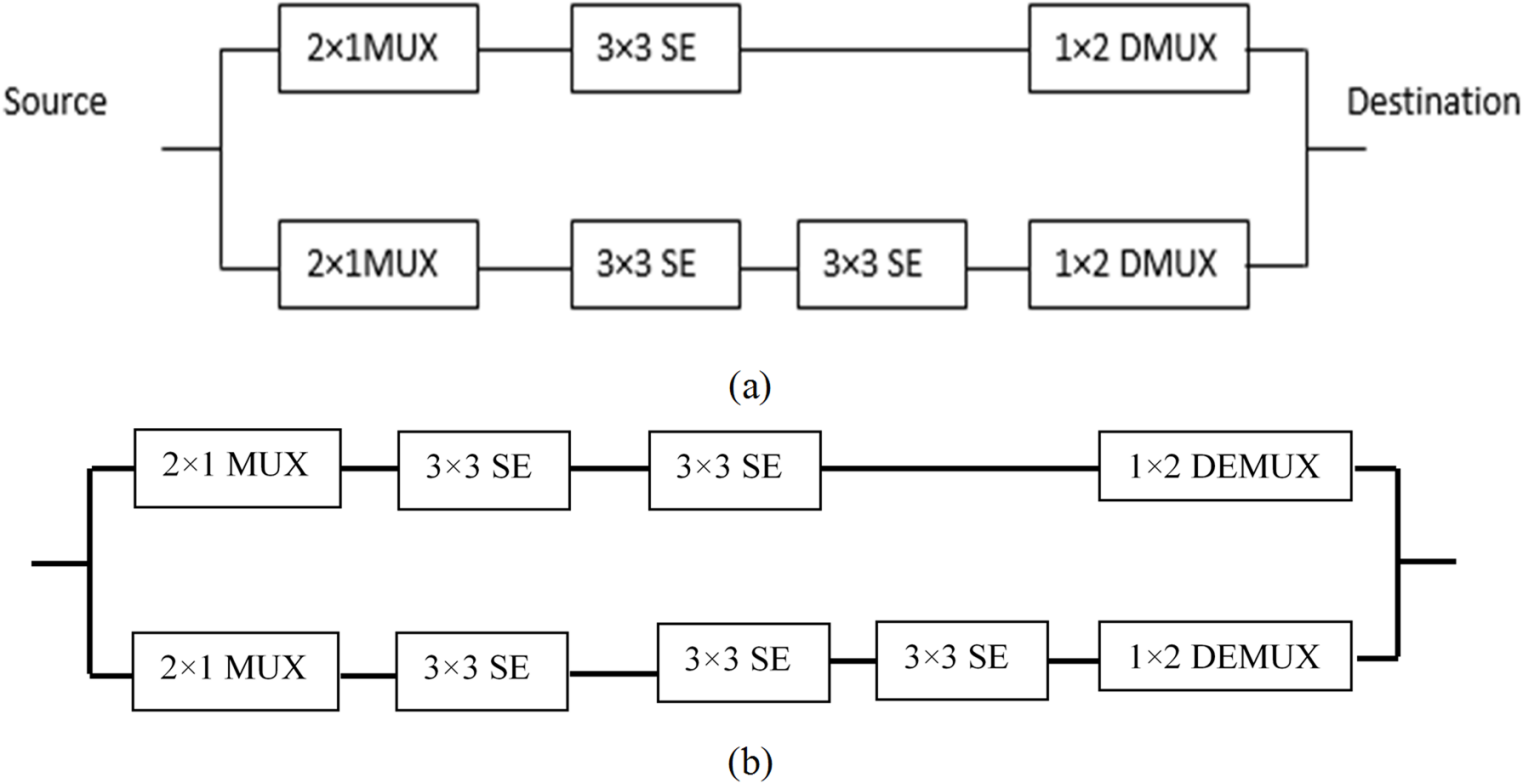
Reliability block diagram of the proposed SSMIN/E Architecture for Terminal Reliability assessment for (**a**) 8 × 8 and (**b**) 16 × 16 Network sizes.

Reliability of a network is always a crucial parameter in the design and evaluation of MINs, specifically for the environments which require efficiency, sustainability and fault tolerance^5,6^. The proposed SSMIN/E architecture aims to offer strong and reliable data transmission. This section presents a detailed reliability analysis of the proposed SSMIN/E architecture, evaluating its fault tolerance capability, improved redundancy, and flexibility against faults. Presence of parallel path between the input and output terminal is always necessary for highly reliable network. An important feature of the proposed SSMIN/E architecture is the formation of totally disjoint parallel paths between its terminal ports. Disjoint paths may be defined as the independent routes that do not share any nodes or links in common. Due to the availability of totally disjoint parallel paths if any fault or contention occurs in one path then also communication between the terminal ports can be established through the other path. Hence, communication would remain uninterrupted even in the presence of faults, making network highly reliable. Also, disjoint parallel paths can be used to transmit multiple data streams simultaneously without the risk of contention or data loss^55–57^. This can in turn increase the network throughput and will correspond to low latency rates. For reliability analysis of proposed SSMIN/E architecture Reliability Block Diagram (RBD) method^58,59^ has been utilized and the RBD of terminal reliability of proposed SSMIN/E architecture has been depicted through Fig. 2.

As shown in the Fig. 2 there are two terminal paths between every source to destination node pair and this RBD is applicable for each S-D node pair. For overall reliability assessment some fundamental presumptions must be taken into account. These presumptions are:

- SEs are either considered to be failed or working no other state is considered such as no SE is assumed to be repairable.
- Links are assumed to be more reliable than system elements i.e. it is assumed that SEs can fail but links can never fail.
- The reliability of a 3 × 3 SE is considered as ‘r’, where 3 × 3 consist of total 9 cross-points and ‘r’ stands for the reliability of all 9 cross-points. Henceforth the reliability of all other system components will be established through this assumption only. For example, 2 × 1 MUX will have reliability of ‘r^2/9^’ and so on.

Where the system reliability ‘r’ can be defined as:

1 $$\:r={\int\:}_{0}^{\infty\:}{e}^{-\lambda\:t}\hspace{0.33em}where\hspace{0.33em}\lambda\:\hspace{0.33em}is\hspace{0.33em}system\hspace{0.33em}failure\hspace{0.33em}rate\hspace{0.33em}assumed\hspace{0.33em}as=1{0}^{-6}\:to\:1{0}^{-7}$$

All SEs are either critical or non-critical, no pre-assumed priority is set on the SEs criticality and these SEs can fail at any random time.

For reliability evaluation through RBD method, two models are used here these are: (1) Series model, as number of system elements are connected in series and (2) Parallel model as two parallel paths are formed for terminal RBD of proposed SSMIN/E architecture^10–15^. For these models the equations for reliability assessment are as follows:

2 $$\:{R}_{series}\:\left(t\right)={r}^{n}\:for\:n\:EquationNumber\:of\:component\:with\:same\:reliability\:r\:$$

3 $$\:{R}_{series}\:\left(t\right)={r}_{1}\times\:{r}_{2}\times\:\dots\:\dots\:{r}_{n}\:for\:component\:with\:different\:reliabilities$$

4 $$\:{R}_{parallel}\:\left(t\right)=1-{\left(1-r\right)}^{n}\:for\:n\:EquationNumber\:of\:component\:with\:same\:reliability\:r$$

5 $$\:{R}_{parallel}\:\left(t\right)=1-\left\{\left(1-{r}_{1}\right)\left(1-{r}_{2}\right)\dots\:\dots\:\dots\:...\left(1-{r}_{n}\right)\right\}\:\:\:for\:component\:with\:different\:reliabilities$$

Hence, the reliability of 8 × 8 SSMIN/E can be calculated using assumption mentioned above with the Eq. (1) to (5). The terminal reliability equation of SSMIN/E is as follows:

Reliability of first path having all components in series is:

6 $$\:{R}_{1}=\:{r}^{2/9}\times\:r\times\:{r}^{2/9}={r}^{13/9}$$

Reliability of second path having all components in series is:

7 $$\:{R}_{2}=\:{r}^{2/9}\times\:r\times\:r\times\:{r}^{2/9}={r}^{22/9}$$

Terminal reliability can now be deduced by combining these two reliability Eq. (6) to (7) as:

8 $$\:R=\:1-\left(\left(1-{r}^{\frac{22}{9}}\right)\left(1-{r}^{\frac{13}{9}}\right)\right)$$

Similarly, for 16 × 16 network size the reliability equation is given in Eq. (9) as:

9 $$\:R=\:1-\left(\left(1-{r}^{\frac{31}{9}}\right)\left(1-{r}^{\frac{22}{9}}\right)\right)$$

For network size ‘N’ the reliability equation can be represented as in Eq. (10):

10 $$\:R=\:1-\left(\left(1-{r}^{\frac{4}{9}}\times\:{r}^{({log}_{2}N-2)}\right)\left(1-{r}^{\frac{4}{9}}\times\:{r}^{({log}_{2}N-1)}\right)\right)$$

In a relatively modern time where massive amount of big data handling and complex calculations are needed, usage of thousands of processors is necessary to accomplish computational horsepower and efficiency to deal with scientific simulations, data analytics, artificial intelligence, and large-scale model simulations^50^. Efficient interconnection of thousands of processing elements (PEs) is required which is critical in high-performance computing and parallel processing^45,46^. The proposed SSMIN/E architecture is capable of being used as an 8 × 8 SE in large-scale parallel processing systems as discussed by the proposed architecture. The proposed SSMIN/E architecture is highly scalable in this case. The inherent architecture of SSMIN/E is a split-plane design, which allows multiple 8 × 8 SSMIN/E based SEs to be combined in a larger network, providing an easy solution for scaling-up the load without any drastic change in the nature of the implementation. This scalability guarantees that, with increasing number of processing elements, the network will maintain high performance and reliability thus rendering proposed SSMIN/E architecture an attractive solution for current high-performance computing environments. Also, the unique mono-stage, multi-path implementation structure of proposed SSMIN/E architecture provides dual-path redundancy for each input/output terminal, makes it have better fault tolerance capability and load balancing capability than others.

While the cost of deploying proposed SSMIN/E architecture as an 8 × 8 SE is slightly higher than that of an 8 × 8 SE based on existing fully crossbar designs, the advantages that it offers clearly validate this investment. The existing fully crossbar 8 × 8 SE provides single path from each input to any other random output port, which may limit throughput under high traffic conditions and is more prone to single points of failures. SSMIN/E has double parallel paths on each input and output terminal. This allows avoiding one of the paths that if one switch fails or congestion happens, the data can be sent to another path, thus this attribute improve the availability and fault tolerance better. The dual-path architecture of SSMIN/E greatly improves the network reliability. In this way, proposed SSMIN/E architecture can have the option of specifying the routing paths according to various fault and congestion conditions and data transfer can become more stable and more reliable. This redundancy is not present in SEs of the traditional fully crossbar 8 × 8, which leaves them open to failures that could cause communication over the network to fail as well. In a large multi-node system this automatic reliability improvement is pretty important, with up-time and consistent performance the most important.

For comparing the quantitative values of computed reliability of proposed SSMIN/E architecture as 8 × 8 SEs against already existing fully crossbar 8 × 8 SEs, can be framed on the basis of assumption taken above. In an 8 × 8 fully crossbar SE with one path between each input output node pair, if one crossbar path having contention, the communication is disrupted. Whereas, in case of proposed SSMIN/E architecture, each connection has two disjoint paths, ensuring higher reliability. The reliability increases as the data contention in any path cannot disturb the communication. Although, the reliability improvement for a single SE is very small but for a large number of SEs, the difference in reliability becomes more pronounced. Even with a small improvement in reliability of individual SE can lead to a noteworthy increase in overall network reliability. For reliability comparison of these two SEs configuration reliability equations are to be framed using assumptions taken as above i.e.

- The reliability of a 3 × 3 SE is *r* (for 3 × 3 = 9 units).

For a fully crossbar 8 × 8 SE (for 8 × 8 = 64 units), the reliability can be expressed using Eq. (9) as given below:

9 $$\:{R}_{fully\:crossbar\:SE}\:\left(t\right)={r}^{64/9}\:$$

For Eq. (9) it has been supposed that the reliability of a 3 × 3 SE is ‘*r*’. The exponent 64/9 is derived on the basis of the number of crossbar connection between every input-output node pairs are required to form a fully crossbar 8 × 8 SE. For SSMIN/E, the reliability equation has been presented in Eq. (8). The computation and comparison of all computed reliability values has been presented and discussed in the proceeding section.

The percentage improvement of SSMIN/E over the traditional 8 × 8 SE can be calculated using Eq. (10). Equation (10) can be used to find the relative increase in reliability provided by proposed SSMIN/E architecture compared to the fully crossbar configuration.

10 $$\:Percentage\:of\:Improvement=\frac{{R}_{SSMIN/E}-{R}_{8\times\:8\:SE}}{{R}_{8\times\:8\:SE}}\times\:100\:\:\:\:\:\:\:\:\:\:\:\:\:$$

## Results and discussion

The evaluated values of terminal reliability ‘R’ of proposed SSMIN/E architecture based on Eq. (8) are as shown in Table 1 with SE reliability ‘r’ varying from 0.99 to 0.90. By analysing network performance data shown in Tables 1 and 2, it is easy to gain deeper understanding of why proposed SSMIN/E architecture is better than prior MINs. With a terminal reliability of 0.99 the proposed SSMIN/E architecture is observed with a fair improvement over the existing MINs with the percentage improvement ranging between 0.0551% and 10.5915%. Meanwhile this suggests that for some networks, while proposed SSMIN/E architecture leads to substantially better performance the gains are more modest. On the other hand, at a TR of 0.90, the magnitude of the improvements is much larger, 4.4550% to 241.2938% improvements.

**Table 1:** TR assessment of the Proposed SSMIN/E Architecture for various values of SE Reliability.

SE reliability = *r*	Terminal reliability of SSMIN/E for network size 8	Terminal reliability of SSMIN/E for network size 16
0.99	0.999650	0.999174
0.98	0.998614	0.996761
0.97	0.996912	0.992854
0.96	0.994562	0.987543
0.95	0.991585	0.980916
0.94	0.987999	0.973057
0.93	0.983823	0.964048
0.92	0.979077	0.953968
0.91	0.973778	0.942893
0.90	0.967946	0.930896

**Table 2:** Terminal reliability and other parametric Comparison of existing variants of shuffle exchange network with the newly proposed SSMIN/E.

Network	Year [ref.]	Path length	Totally disjoint paths	TR		Percentage improvement of SSMIN/E over existing MINs	
				0.99	0.90	0.99	0.90
SEN-	2016 [35, 36]	2	2	0.9991	0.9266	0.0551%	4.4550%
SEN	1989 [19]	3	0	0.9703	0.729	3.0274%	32.8258%
SEN+	2008 [13]	4	0	0.9797	0.7808	2.0362%	23.9821%
SEN + 2	2008 [13]	5	0	0.9797	0.7782	2.0362%	24.4181%
SHSEN	2014 [25]	4	2	0.999	0.9173	0.0651%	5.5153%
ABN	1994 [34]	2 (varying)	0	0.9347	0.4295	6.9453%	125.3675%
BENES	2002 [20]	5	0	0.9699	0.7004	3.0661%	38.2276%
REP-MIN	2006 [26]	3	2	0.9991	0.9266	0.0551%	4.4550%
EGN	1988 [44]	2	3	0.9227	0.4305	8.3440%	124.8661%
IEGN	2014 [6]	2 (varying)	3	0.9039	0.2836	10.5915%	241.2938%
Pars	2015 [7]	2	3	0.9406	0.4924	6.2734%	96.6193%
ASEN	1987 [9, 46]	2 (varying)	2	0.999	0.9037	0.0651%	7.1066%
GIN	1984 [19]	4	0	0.9606	0.6561	4.065168%	47.53%
GIN-Minus	2015/2019 [8, 12]	3	2	0.9996	0.9602	0.005%	0.806%
SEGIN	2017 [33]	4	0	0.9794	0.7631	2.0676%	26.84%
SEGIN-Minus	2020 [21]	3	2	0.9847	0.8272	1.518229%	17.01475%
4-DGIN	2014 [32]	4	0	0.9832	0.8270	1.673108%	17.0431%
4D-GIN-1	2020 [59]	4	1	--	0.80936	--	19.594%
4D-GIN-2	2020 [59]	4	1	--	0.80955	--	19.56593%
4D-GIN-3	2020 [59]	4	1	--	0.80955	--	19.56593%
SEGLNIN	2019 [60]	4	1	--	0.800442	--	20.92644%
RGN	2023 [61]	2	2	0.9996	--	0.005%	--
EASEN	2024 [62]	2 (varying)	2	0.9990	0.8877	0.065067%	12.61124%
FTGM-1	2023 [15]	3	4	0.9998	0.9697	-0.015%	-0.18088%
SSMIN/E	Proposed	1 (varying)	2	0.999650	0.967946	--	--

Although FTGM performs slightly better than that of SSMIN/E in terms of TR but it has a higher path length, hardware complexity and cost than that of proposed SSMIN/E. Hence, the proposed SSMIN/E architecture provides a more pragmatic and balanced solution providing near-identical fault tolerance with significantly less path length, hardware complexity, and implementation cost, placing it as an appropriate solution for scalable and cost-sensitive applications such as NoCs and sensor networks. This improvement is significant which means the proposed SSMIN/E architecture performs very well under high traffic environments resulting in effective congestion reduction and massive data flow capacity improvement. The path length and disjoint paths metrics also underline the benefits of SSMIN/E. It has a path length of 1 which means that routing is very efficient. It also has 2 disjoint paths, which increases the fault tolerance and reliability. The other networks have path lengths of between 2 and 5 and 0 to 3 disjoint paths, demonstrating significant diversity in design and capability. SSMIN/E compares favourably with respect to its ability to generate TR values ≥ 0.99, which are observed across network comparisons at both 0.99 and 0.90 TR. Although IEGN and ABN also yield better results for the high traffic conditions, they fall short of the flexibility of SSMIN/E. These results taken together identify SSMIN/E as an effective network design in terms of short path lengths (low PL) to reach different servers from a user combined with multiple disjoint paths, and high TR values under heavy traffic which underscores its robustness and effectiveness.

**Fig. 3:**
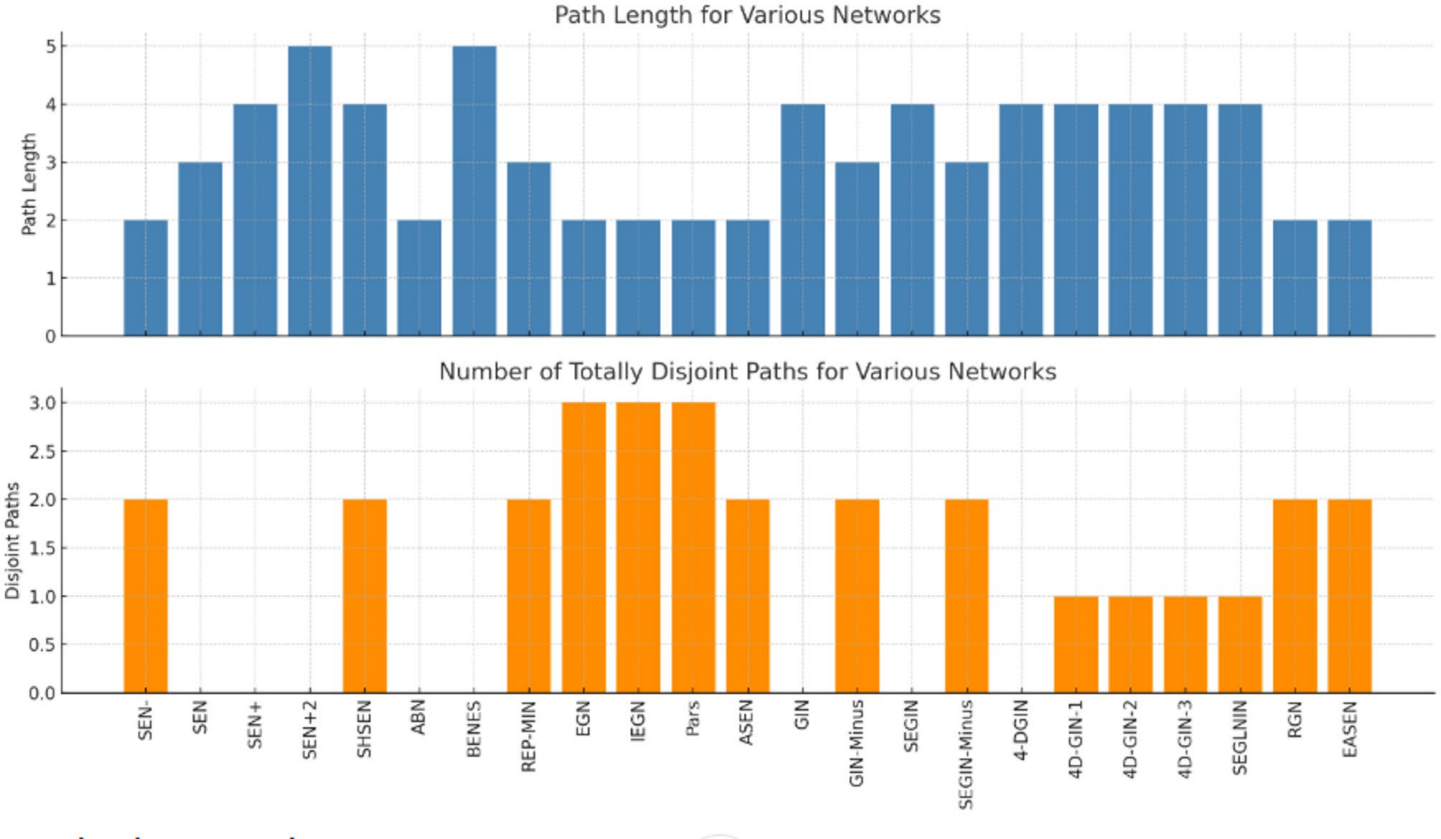
Bar graph of path length and disjoint paths of the Proposed SSMIN/E Architecture and other existing MINs.

**Fig. 4:**
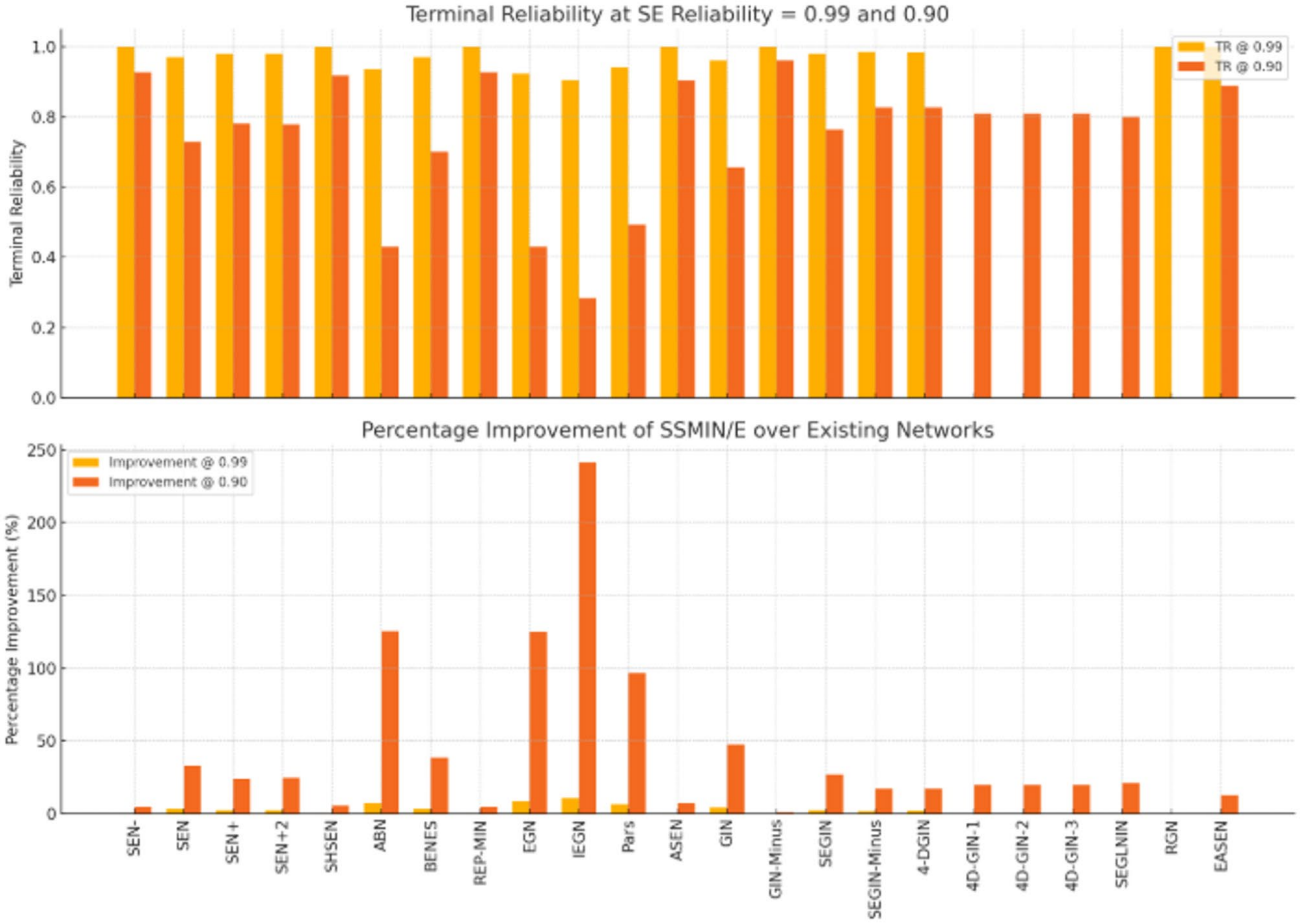
TR of the Proposed SSMIN/E Architecture over other studied MINS for SE Reliability ‘r’ = 0.90 and 0.99 and its percentage improvement for both SE Reliabilities.

The visualization of all these achieved results is shown in bar graphs represented by Figs. 3 and 4. The Fig. 3 displays the path lengths for every network. SSMIN/E with a path length of 1 and other MINs with a path length of 2 to 5 as path lengths differs for every network. Also, it shows the count of disjoint paths. Disjoint paths are 2 for SSMIN/E and of the other networks it ranges from 0 to 3. The plot displayed in Fig. 4 illustrates the percentage improvement of SSMIN/E over the existing MINs at SE reliability ‘r’ = 0.99 and 0.90. We see improvement ranging from the single digits to multiple digits. Figure 4 also shows the TR improvement values of SSMIN/E at 0.99 and 0.90 for each network. It shows that SSMIN/E always follows dominating TR values. These plots explain in detail the comparative performance of SSMIN/E with the MINs under higher traffic conditions demonstrating its efficiency.

Further, the reliability comparison has also been made between 8 × 8 fully crossbar SE and SSMIN/E as 8 × 8 SE. Table 3 shows the computed values of reliability of both SE with respect to ‘r’ and the percentage improvement of SSMIN/E over existing 8 × 8 SE.

**Table 3:** Reliability and percentage improvement assessment of the Proposed SSMIN/E Architecture versus existing 8 × 8 SE for various Reliability values of 3 × 3 SE.

*r* (as assumed for 3 × 3 SE)	*R*_fully crossbar 8 × 8 SE_	*R*_SSMIN/E_	Percentage Improvement (%)
0.90	0.472730	0.967946	104.76
0.91	0.511374	0.973778	90.42
0.92	0.552702	0.979077	77.14
0.93	0.596869	0.983823	64.83
0.94	0.644035	0.987999	53.41
0.95	0.694369	0.991585	42.80
0.96	0.748047	0.994562	32.95
0.97	0.805253	0.996912	23.80
0.98	0.866179	0.998614	15.29
0.99	0.931025	0.999650	7.37

**Fig. 5:**
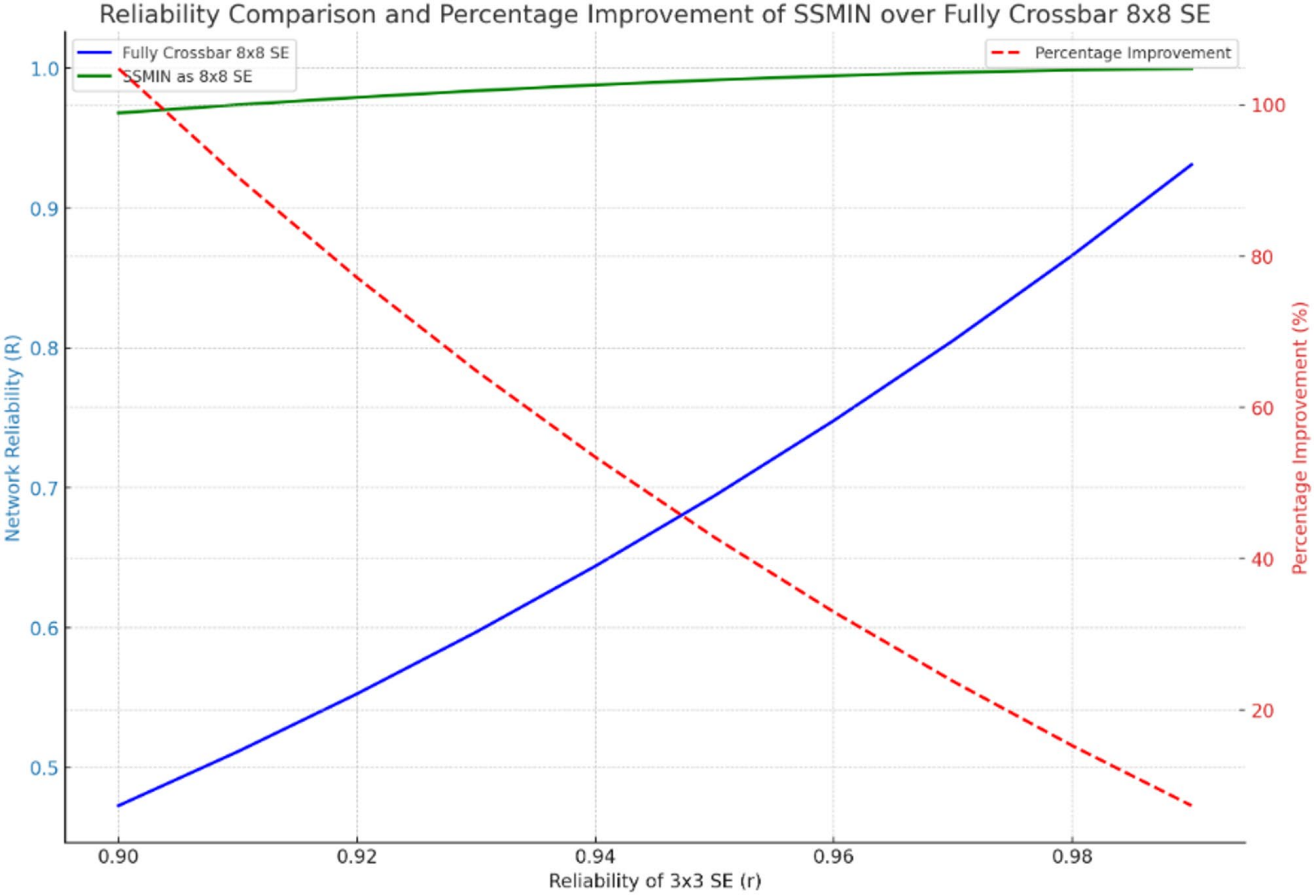
Graphical Comparison of Reliability and improved percentage of the Proposed SSMIN/E Architecture over traditional 8 × 8 SE.

Figure 5 describes the reliability performance 8 × 8 fully crossbar SE and proposed SSMIN/E architecture as 8 × 8 SE as ‘r’ varies from 0.90 to 0.99. For ‘r’ less than 0.9, reliability of an 8 × 8 SE tends to reduce rapidly. The reason for this is that it has single connection between each input to output node. The reliability R of 8 × 8 fully crossbar SE is found approximately as 0.80826 for *r*=0.9, which means that vulnerability to faults increases to a significant extent as ‘*r*’ decreases. The proposed SSMIN/E architecture is always reliably higher within the given ranges of ‘*r*’ values. That is because proposed SSMIN/E architecture has the redundancy due to its dual-path architecture that will reroute data when it encounters a contention in the switch terminal.

From Fig. 5, it is obvious that the network reliability of the proposed SSMIN/E architecture under *r*=0.9 is about 0.84, which indicates the robustness and higher fault tolerance of this network. The reliability comparison shows that as an 8 × 8 SE, proposed SSMIN/E architecture provides the highest fault tolerance and reliability when compared with the conventional full crossbar 8 × 8 SE. With decreasing *r*, the performance gap widens, making proposed SSMIN/E architecture more appealing for packet switched broadband communication system. This has also been proven by the percentage improvement achieved by proposed SSMIN/E architecture over the existing 8 × 8 SE. This enhanced reliability is essential when thousands of PEs form a large-scale environment to handle massive amounts of data, to keep performance and uptime consistent.

The proposed SSMIN/E architecture provides a fault-tolerant, cost-effective solution to conventional multistage networks such as Gamma, especially in applications where low latency and low hardware complexity are critical. Unlike Gamma networks, which involve numerous switching stages and high cross-point counts, proposed SSMIN/E architecture provides redundancy through a single stage of larger-sized SEs with the addition of MUX/DEMUX units. To analyse its cost in terms of hardware, the component-based costing strategy is utilized which has been a normal practice in literature commonly applied in interconnection network research. According to this strategy, the network cost is approximated depending upon the number of components category utilized and the corresponding cross-points. For cost evaluation of proposed SSMIN/E architecture, the following method has been used which is suggested in many existing papers [59-62]:

- In each stage 8 inputs and 8 outputs use eight 2 × 1 MUX and 8 1 × 2 DEMUX. Each of which contributes 2 cross-points, yielding:
  - Total cost for MUX/DEMUX stage = 2 × 8 × 2 = 4 × 8 cross-points.
- Switching Elements (SEs): The single-stage switch uses four 3 × 3 SEs for 8 × 8 configurations. Each 3 × 3 SE has 9 cross-points:
  - Total switching cost = 4 × 9 = 36 cross-points.

Thus, an 8 × 8 proposed SSMIN/E has:

- MUX/DEMUX cost = 4 × 8 = 32.
- Switching cost = 36.
- Total hardware cost = 68 cross-points.

The Cost evaluation of SSMIN/E for higher Network sizes = 2×N + B_SE_+B_MUX/DEMUX_.

Where:

- For block of MUX and DEMUX B_MUX/DEMUX_ = 2×(N MUX + N DEMUX) = 4 N.
- For block of SEs B_SE_=N/2 3 × 3 SE per stages (where stages are log_2_N-2), for example for 8 × 8 network size the stages of SEs used are log_2_8 − 2 = 1.

Thus, Total Cost of proposed SSMIN/E = 4 *N* + 9×N/2 × log_2_N-2 = 4 *N* + 4.5 N(log_2_N-2).

Gamma networks require multiple stages of SEs (e.g., for log_2_*N* + 1, ‘N’ SEs are required where input stage is made up of N numbers of 1 × 3 SEs and output stage consist of N numbers of 3 × 1 SEs and rest log_2_N-1 stages consist of N numbers of 3 × 3 SEs) and other networks are similar too. In comparison to these existing MIN SSMIN/E immensely decreases both the component count and total cost of interconnection, while retaining dual-path fault tolerance. The SSMIN/E architecture is further evaluated using the Reliability-Cost Ratio (RCR) to compare network architectures reasonably. It measures the amount of reliability per unit cost that is achieved, and it can be used to find cost-effective fault-tolerant designs (RCR = Terminal reliability/Cost). Although terminal reliability by itself indicates network reliability, it does not capture the cost that goes into it. Two networks could have the same TR but employ far greater hardware. The RCR (1) encourages cost-conscious design choices, (2) facilitates equitable comparison between complex and compact topologies, and (3) assists designers in making optimal trade-offs between reliability and utilization. In embedded and high-performance systems such as sensor networks, NoCs, HPC clusters etc., this measure is critical to choose networks not only that are reliable, but also feasible and scalable.

For more clarity on this aspect the cost comparison all studied MINs in Table 2 has been given in the Table 4, which clearly depicts the better performance of SSMIN/E as 8 × 8 SE. the Table 4 also gives the cost reliability ratio for clear validation of SSMIN/E superiority over other studied MINs. Since SEN have a lower cost than SSMIN/E and only slightly lower reliability, their reliability-cost ratio (RCR) is higher, making them more cost-efficient overall in the 8 × 8 configuration. The negative percentage improvement of SSMIN/E indicates that although it provides stronger fault tolerance and architectural advantages, it does so at a higher cost per unit of reliability compared to the more lightweight SEN MIN, which is non fault tolerant network. Therefore, SEN is not preferable in reliable cost-constrained scenarios where moderate reliability suffices.

**Table 4:** Cost comparison of various MINs under study with the proposed SSMIN/E.

Network	Cost computation for 8 × 8 network size	Reliability cost ratio	Percentage improvement of SSMIN/E (in %)
SEN	48	0.015188	-6.2749
SEN+	64	0.0122	16.67623
SEN + 2	80	0.009728	46.33256
SHSEN	128	0.007166	98.62815
ABN	200	0.002148	562.8405
REP-MIN	96	0.009652	47.47593
EGN	96	0.004484	217.4244
IEGN	156	0.001818	682.9979
Pars	120	0.004103	246.9009
ASEN	84	0.010758	32.31139
GIN	192	0.003417	316.556
GIN-Minus	152	0.006317	125.3326
SEGIN	80	0.009539	49.22815
SEGIN-Minus	80	0.01034	37.66441
4-DGIN	160	0.005169	175.3954
4D-GIN-1	160	0.005059	181.3976
4D-GIN-2	160	0.00506	181.3316
4D-GIN-3	160	0.00506	181.3316
SEGLNIN	100	0.008004	77.833
EASEN	104	0.008536	66.7667
Proposed SSMIN/E	68	0.014235	--

**Fig. 6:**
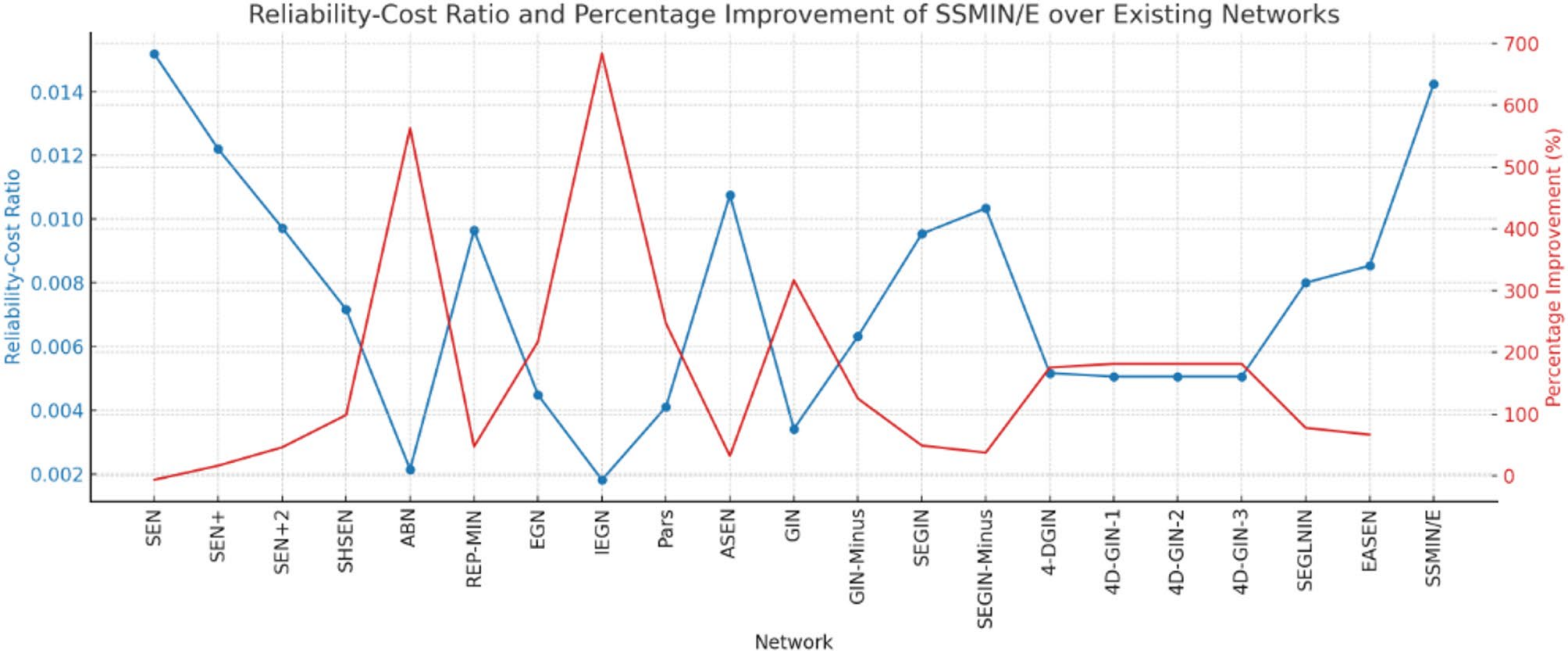
Line graph depicting the RCR and percentage improvement of the Proposed SSMIN over other MINs.

Figure 6 depicts that network like IEGN, ABN, and Pars have very low RCR values though these networks are inefficient as per the cost is concerned. Whereas, SSMIN/E maintains a strong RCR, even outperforming some variants with much higher cost. Also, the percentage improvements are substantial for older or less optimized topologies.

Thus, the proposed SSMIN/E architecture enables increased network throughput by enabling multiple data streams to be transmitted in parallel. This proved to be a huge advantage in high-performance computing environments where large amount of data has to be analysed and transferred at a very high speed. The use of two parallel paths reduces latency by eliminating the data contentions and ensuring the delivery of data packets to their destinations immediately which is the second advantage of using the proposed SSMIN/E for high-speed computations. For real-time applications like financial trading, video streaming, etc., this highly reduced latency is a boon. The proposed SSMIN/E architecture has proved itself as a scalable MIN for large networks which makes it suitable for many use cases and environments. The smaller networks can easily be scaled up to larger sizes with the use of proposed SSMIN/E as 8 × 8 SE. This is a very useful advantage especially for data centres and cloud computing where network resources and input/output nodes can vary frequently. Another very critical advantage of proposed SSMIN/E architecture is the fault tolerance which is provided because of distinct multiple paths in parallel to ensure that the network perform its desired function even in the presence of failure of hardware, connections or other issues. Such dependability is imperative to the provision of nonstop service and, eliciting through the roof demands for the contemporary users.

As the proposed SSMIN/E architecture possesses two totally disjoint paths, it allows SSMIN/E SE atleast single fault/node contention tolerant. Also, the computed reliability values of SSMIN/E as an 8 × 8 SE consistently exceeds than that of an existing fully crossbar 8 × 8 SE at most reliability levels of the ‘r’ (3 × 3 SE reliability). For example, at *r* = 0.90, SSMIN/E reliability is ~ 0.968, compared to the fully crossbar SE whose reliability is only 0.473, a significant 104.76% improvement. SSMIN/E achieves a reliability of 0.9997 compared to 0.931 in the fully crossbar SE even at a much lower reliability number, *r* of 0.99 equivalent to a 7.37% reduction. SSMIN/E keeps this high-performance advantage constant and demonstrates that it is more fault tolerant and robust against the failures and the expensive pre-emption in the large-scale high-performance computing environments (HPC). In conclusion, this architecture and switching method have shown significant improvement for multipath interconnection networks and provide an effective solution for the real-time data transmission problems. The proposed SSMIN/E architecture can be tuned to provide an effective and scalable solution to data transmissions problem needed in the era of big data. Using 3 × 3 crossbar switches that provide modular flexibility and with special emphasis on MUX/DEMUX this network provides high utilization, low latency, and good fault tolerance support. This technology provides support for different routes that data packets can take simultaneously, without preventing or affecting one of the others on the network pathways, which makes use of the redundancy of network resources to maintain reliability. Moreover, the network is scalable to suit most applications and environments, from high-performance computing to hyperscale data centres.

## Conclusion and future scope

The proposed SSMIN/E architecture demonstrates optimization in routing capability, reliability and cost within a one-stage setup, presenting a realistic solution that fills the gap between highly redundant topologies and hardware-efficient single-path networks. The proposed SSMIN/E architecture elements include four 3 × 3 SE subunits which contain eight 2 × 1 multiplexers and 1 × 2 demultiplexers to create two parallel paths between every source and destination link. The new design method improves system reliability because it allows multiple faults to be handled while maintaining simple network operations which require less processing power. The proposed SSMIN/E architecture has been evaluated for its reliability and its improvements over current MINs. Based on the evaluation, the proposed SSMIN/E architecture shows significantly better quantitative advantages over the prior MINs. With SE reliability of 0.99, the improvement in proposed SSMIN/E architecture performance falls within the range of 0.0551% to 10.5915%, while, achieving an impressive gain as high as 241.2938% especially at higher traffic conditions, most notably at SE reliability of 0.80 it shows 4.4550% to an impressive 241.2938%. These results prove the efficiency of SSMIN/E over its counterparts. Moreover, the proposed SSMIN/E architecture has the shortest path length, i.e. one path length and has two disjoint paths, which indicates improved routing efficiency and optimal reliability performance, respectively. In terms of network architecture, proposed SSMIN/E architecture is superior to other architectures, as indicated by the high TR values, its short path lengths, and substantial performance gains. The proposed SSMIN/E architecture mitigates the problem of existing 8 × 8 SE which include a fully cross-bar network which is inefficient and does not provide multiple paths and the packet is either dropped or buffered in case of contention at a specified output node.

We believe that the potential of the proposed SSMIN/E architecture can be harnessed further. Scalability can be increased to support the growing data traffic of larger and larger Network sizes while maintaining high performance. There is also immense opportunity to identify its integration with emerging technologies on modern edge platforms such as 5G/6G, IoT, AI for new applications requiring both high performance and low levels of latency.

## Data Availability

Not applicable.
